# Correction: Understanding the impact of moxifloxacin on immune function: Findings from cytokine analyses and immunological assays in mice

**DOI:** 10.1371/journal.pone.0326272

**Published:** 2025-06-10

**Authors:** The PLOS One Staff

There are errors in the author affiliations. The correct affiliations are as follows:

Mehmood Ahmad^1,2^, Naeem Rasool^2^, Rana Muhammad Zahid Mushtaq^3^, Sammina Mahmood^4^, Abdur Rauf Khalid^5^, Waqas Ahmad^6,7^, Bilal Mahmood Beg^2^, Mostafa A. Abdel Maksoud^8^, Abdul Aziz Alamri^8^, Adeel Sattar^2^

1 Department of Pharmacology and Toxicology, Faculty of Veterinary and Animal Sciences, The Islamia University of Bahawalpur, Bahawalpur, Pakistan, 2 Department of Pharmacology and Toxicology, University of Veterinary and Animal Sciences, Lahore, Pakistan, 3 Division of Infection Medicine, College of Medicine, The University of Edinburgh, Edinburgh, United Kingdom, 4 Department of Botany, Division of Science and Technology, Bank Road Campus, University of Education, Lahore, Pakistan, 5 Department of Livestock and Poultry Production, Faculty of Veterinary Sciences, Bahauddin Zakariya University, Multan, Pakistan, 6 Department of Pathology, University of Veterinary and Animal Sciences, Lahore, Pakistan, 7 Livestock and Diary Development Department, Government of Punjab, Lahore, Pakistan, 8 Biochemistry Department, College of Science, King Saud University, Riyadh, Saudi Arabia

The Funding statement for this article is incorrect. The correct Funding statement is as follows: The authors extend their appreciation to the Researchers Supporting Project number (RSPD2025R552) King Saud University, Riyadh, Saudi Arabia.

The publisher apologizes for the errors.
