# Supplementary material for: Understanding the impact of moxifloxacin on immune function: Findings from cytokine analyses and immunological assays in mice
Source: PLoS One. 2025 May 6;20(5):e0321961. doi: 10.1371/journal.pone.0321961 (PMC12054891; doi:10.1371/journal.pone.0321961)
Supplement: S1 Data — (DOCX) [file pone.0321961.s001.docx]

**Statistical Analyses Data:**

K AND ALPHA

**One-way ANOVA: K (Carbon Clearance Rate) versus Group**

**Method**

| Null hypothesis | All means are equal |
| --- | --- |
| Alternative hypothesis | Not all means are equal |
| Significance level | α = 0.05 |

*Equal variances were assumed for the analysis.*

**Factor Information**

| **Factor** | **Levels** | **Values** |
| --- | --- | --- |
| Group | 4 | Control group, Moxifloxacin 15mg/kg, Moxifloxacin 3.75mg/kg, Moxifloxacin 7.5mg/kg |

**Analysis of Variance**

| **Source** | **DF** | **Seq SS** | **Contribution** | **Adj SS** | **Adj MS** | **F-Value** | **P-Value** |
| --- | --- | --- | --- | --- | --- | --- | --- |
| Group | 3 | 0.000378 | 51.42% | 0.000378 | 0.000126 | 5.64 | 0.008 |
| Error | 16 | 0.000358 | 48.58% | 0.000358 | 0.000022 |  |  |
| Total | 19 | 0.000736 | 100.00% |  |  |  |  |

**Model Summary**

| **S** | **R-sq** | **R-sq(adj)** | **PRESS** | **R-sq(pred)** |
| --- | --- | --- | --- | --- |
| 0.0047275 | 51.42% | 42.31% | 0.0005587 | 24.09% |

**Means**

| **Group** | **N** | **Mean** | **StDev** | **95% CI** |
| --- | --- | --- | --- | --- |
| Control group | 5 | 0.022386 | 0.002202 | (0.017904, 0.026868) |
| Moxifloxacin 15mg/kg | 5 | 0.01331 | 0.00692 | (0.00883, 0.01779) |
| Moxifloxacin 3.75mg/kg | 5 | 0.02379 | 0.00462 | (0.01931, 0.02827) |
| Moxifloxacin 7.5mg/kg | 5 | 0.01600 | 0.00391 | (0.01152, 0.02048) |

*Pooled StDev = 0.00472746*

**Tukey Pairwise Comparisons**

**Grouping Information Using the Tukey Method and 95% Confidence**

| **Group** | **N** | **Mean** | **Grouping** | |
| --- | --- | --- | --- | --- |
| Moxifloxacin 3.75mg/kg | 5 | 0.02379 | A |  |
| Control group | 5 | 0.022386 | A |  |
| Moxifloxacin 7.5mg/kg | 5 | 0.01600 | A | B |
| Moxifloxacin 15mg/kg | 5 | 0.01331 |  | B |

*Means that do not share a letter are significantly different.*


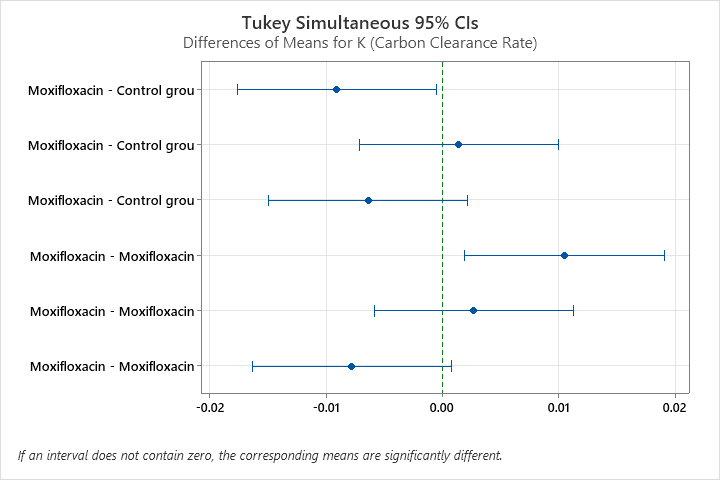


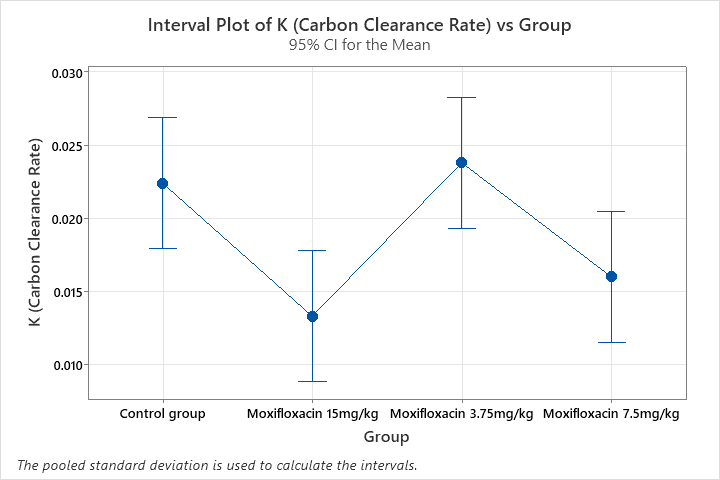


K AND ALPHA

**One-way ANOVA: α (Phagocytic index) versus Group**

**Method**

| Null hypothesis | All means are equal |
| --- | --- |
| Alternative hypothesis | Not all means are equal |
| Significance level | α = 0.05 |

*Equal variances were assumed for the analysis.*

**Factor Information**

| **Factor** | **Levels** | **Values** |
| --- | --- | --- |
| Group | 4 | Control group, Moxifloxacin 15mg/kg, Moxifloxacin 3.75mg/kg, Moxifloxacin 7.5mg/kg |

**Analysis of Variance**

| **Source** | **DF** | **Seq SS** | **Contribution** | **Adj SS** | **Adj MS** | **F-Value** | **P-Value** |
| --- | --- | --- | --- | --- | --- | --- | --- |
| Group | 3 | 4.751 | 45.58% | 4.751 | 1.5838 | 4.47 | 0.018 |
| Error | 16 | 5.674 | 54.42% | 5.674 | 0.3546 |  |  |
| Total | 19 | 10.426 | 100.00% |  |  |  |  |

**Model Summary**

| **S** | **R-sq** | **R-sq(adj)** | **PRESS** | **R-sq(pred)** |
| --- | --- | --- | --- | --- |
| 0.595508 | 45.58% | 35.37% | 8.86573 | 14.96% |

**Means**

| **Group** | **N** | **Mean** | **StDev** | **95% CI** |
| --- | --- | --- | --- | --- |
| Control group | 5 | 6.5251 | 0.1651 | (5.9606, 7.0897) |
| Moxifloxacin 15mg/kg | 5 | 5.501 | 0.982 | (4.936, 6.065) |
| Moxifloxacin 3.75mg/kg | 5 | 6.782 | 0.590 | (6.217, 7.346) |
| Moxifloxacin 7.5mg/kg | 5 | 6.064 | 0.282 | (5.500, 6.629) |

*Pooled StDev = 0.595508*

**Tukey Pairwise Comparisons**

**Grouping Information Using the Tukey Method and 95% Confidence**

| **Group** | **N** | **Mean** | **Grouping** | |
| --- | --- | --- | --- | --- |
| Moxifloxacin 3.75mg/kg | 5 | 6.782 | A |  |
| Control group | 5 | 6.5251 | A | B |
| Moxifloxacin 7.5mg/kg | 5 | 6.064 | A | B |
| Moxifloxacin 15mg/kg | 5 | 5.501 |  | B |

*Means that do not share a letter are significantly different.*


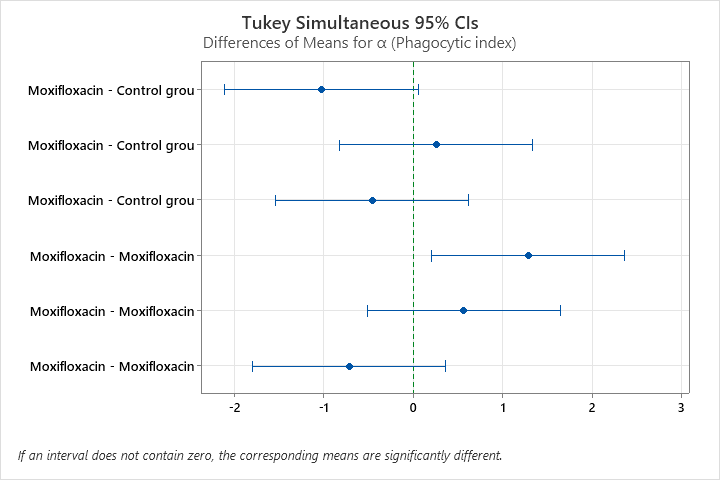


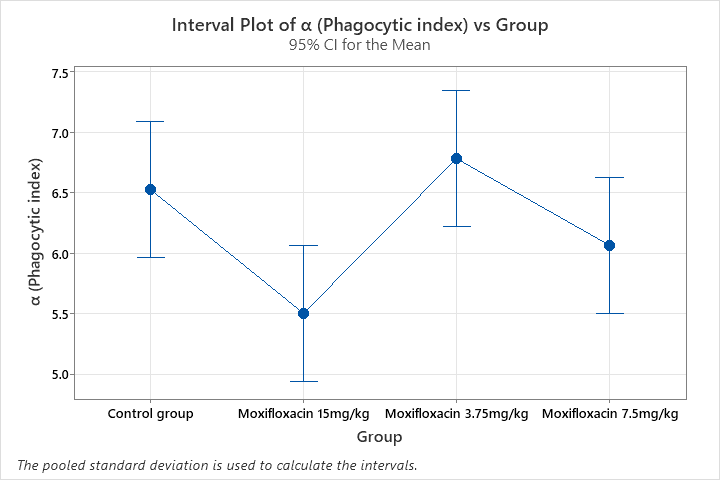


UNKNOWN WORKSHEET

**One-way ANOVA: control, MXF 4µg/ml, MXF 8µg/ml, MXF 16µg/ml, MXF 32µg/ml, MXF 64µg/ml**

**Method**

| Null hypothesis | All means are equal |
| --- | --- |
| Alternative hypothesis | Not all means are equal |
| Significance level | α = 0.05 |

*Equal variances were assumed for the analysis.*

**Factor Information**

| **Factor** | **Levels** | **Values** |
| --- | --- | --- |
| Factor | 6 | control, MXF 4µg/ml, MXF 8µg/ml, MXF 16µg/ml, MXF 32µg/ml, MXF 64µg/ml |

**Analysis of Variance**

| **Source** | **DF** | **Seq SS** | **Contribution** | **Adj SS** | **Adj MS** | **F-Value** | **P-Value** |
| --- | --- | --- | --- | --- | --- | --- | --- |
| Factor | 5 | 141.77 | 78.04% | 141.77 | 28.354 | 8.53 | 0.001 |
| Error | 12 | 39.89 | 21.96% | 39.89 | 3.324 |  |  |
| Total | 17 | 181.66 | 100.00% |  |  |  |  |

**Model Summary**

| **S** | **R-sq** | **R-sq(adj)** | **PRESS** | **R-sq(pred)** |
| --- | --- | --- | --- | --- |
| 1.82322 | 78.04% | 68.89% | 89.7514 | 50.59% |

**Means**

| **Factor** | **N** | **Mean** | **StDev** | **95% CI** |
| --- | --- | --- | --- | --- |
| control | 3 | 24.129 | 1.713 | (21.836, 26.423) |
| MXF 4µg/ml | 3 | 23.319 | 1.050 | (21.026, 25.613) |
| MXF 8µg/ml | 3 | 25.78 | 2.93 | (23.49, 28.07) |
| MXF 16µg/ml | 3 | 28.04 | 1.83 | (25.75, 30.34) |
| MXF 32µg/ml | 3 | 30.826 | 1.415 | (28.533, 33.120) |
| MXF 64µg/ml | 3 | 22.965 | 1.406 | (20.672, 25.259) |

*Pooled StDev = 1.82322*

**Tukey Pairwise Comparisons**

**Grouping Information Using the Tukey Method and 95% Confidence**

| **Factor** | **N** | **Mean** | **Grouping** | | |
| --- | --- | --- | --- | --- | --- |
| MXF 32µg/ml | 3 | 30.826 | A |  |  |
| MXF 16µg/ml | 3 | 28.04 | A | B |  |
| MXF 8µg/ml | 3 | 25.78 |  | B | C |
| control | 3 | 24.129 |  | B | C |
| MXF 4µg/ml | 3 | 23.319 |  | B | C |
| MXF 64µg/ml | 3 | 22.965 |  |  | C |

*Means that do not share a letter are significantly different.*


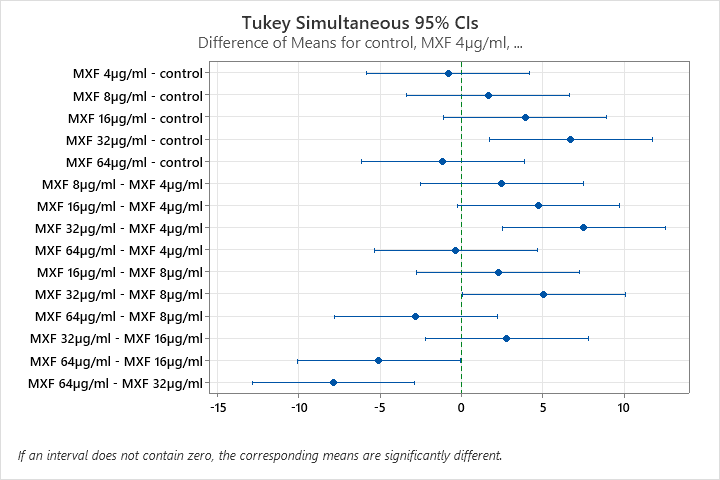


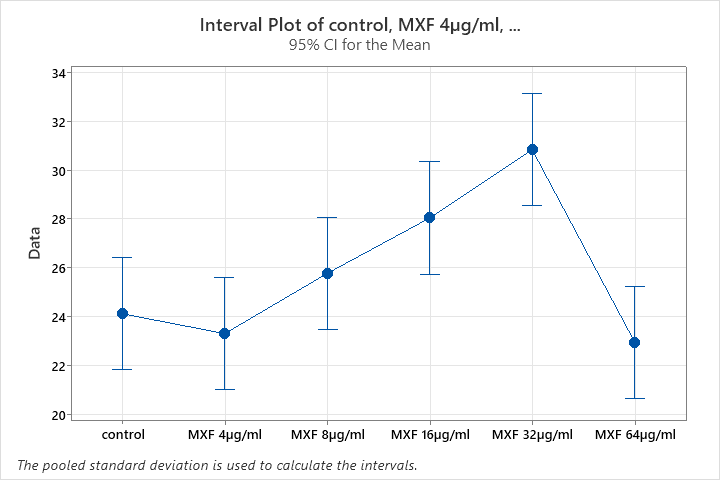


MICE LETHALITY

**Tabulated Statistics: group no., Response of mice**

* NOTE * Fisher’s exact test is available only for 2 x 2 tables.

**Rows: group no.   Columns: Response of mice**

|  | **alive** | **dead** | **All** |
| --- | --- | --- | --- |
|  |  |  |  |
| MXF 15 mg/kg | 1 | 4 | 5 |
| MXF 3.75 mg/kg | 3 | 2 | 5 |
| MXF 7.5 mg/kg | 2 | 3 | 5 |
| Negative Control | 5 | 0 | 5 |
| Positive Control | 0 | 5 | 5 |
| All | 11 | 14 | 25 |

*Cell Contents
      Count*

**Chi-Square Test**

|  | **Chi-Square** | **DF** | **P-Value** |
| --- | --- | --- | --- |
| Pearson | 12.013 | 4 | 0.017 |
| Likelihood Ratio | 15.832 | 4 | 0.003 |

*10 cell(s) with expected counts less than 5.*

MICE LETHALITY

**Binary Logistic Regression: Response of mice versus group no.**

* WARNING * The model could not be fit properly. Maximum likelihood estimates of parameters
may not exist due to quasi-complete separation of data points. The results might not be
reliable. Please refer to help for more information about quasi-complete separation.

**Method**

| Link function | Logit |
| --- | --- |
| Categorical predictor coding | (1, 0) |
| Rows used | 25 |

**Response Information**

| **Variable** | **Value** | **Count** |  |
| --- | --- | --- | --- |
| Response of mice | dead | 14 | (Event) |
|  | alive | 11 |  |
|  | Total | 25 |  |

**Regression Equation**

| P(dead) | | | = | exp(Y')/(1 + exp(Y')) |  |
| --- | --- | --- | --- | --- | --- |
| Y' | = | 1.39 + 0.0 group no._MXF 15 mg/kg - 1.79 group no._MXF 3.75 mg/kg - 0.98 group no._MXF 7.5 mg/kg - 15 group no._Negative Control + 12 group no._Positive Control | | | |

**Coefficients**

| **Term** | **Coef** | **SE Coef** | **Z-Value** | **P-Value** | **VIF** |
| --- | --- | --- | --- | --- | --- |
| Constant | 1.39 | 1.12 | 1.24 | 0.215 |  |
| group no. |  |  |  |  |  |
| MXF 3.75 mg/kg | -1.79 | 1.44 | -1.24 | 0.214 | 1.56 |
| MXF 7.5 mg/kg | -0.98 | 1.44 | -0.68 | 0.497 | 1.56 |
| Negative Control | -15 | 239 | -0.06 | 0.950 | 1.00 |
| Positive Control | 12 | 239 | 0.05 | 0.959 | 1.00 |

**Odds Ratios for Categorical Predictors**

| **Level A** | **Level B** | **Odds Ratio** | **95% CI** |
| --- | --- | --- | --- |
| group no. |  |  |  |
| MXF 3.75 mg/kg | MXF 15 mg/kg | 0.1667 | (0.0098, 2.8213) |
| MXF 7.5 mg/kg | MXF 15 mg/kg | 0.3750 | (0.0222, 6.3480) |
| Negative Control | MXF 15 mg/kg | 0.0000 | (0.0000, 2.10318E+197) |
| Positive Control | MXF 15 mg/kg | 194808.4666 | (0.0000, 1.27706E+209) |
| MXF 7.5 mg/kg | MXF 3.75 mg/kg | 2.2500 | (0.1792, 28.2539) |
| Negative Control | MXF 3.75 mg/kg | 0.0000 | (0.0000, 1.25976E+198) |
| Positive Control | MXF 3.75 mg/kg | 1.16885E+06 | (0.0000, 7.64931E+209) |
| Negative Control | MXF 7.5 mg/kg | 0.0000 | (0.0000, 5.59892E+197) |
| Positive Control | MXF 7.5 mg/kg | 519489.2443 | (0.0000, 3.39969E+209) |
| Positive Control | Negative Control | 6.07205E+11 | (0.0000, 1.04809E+300) |

*Odds ratio for level A relative to level B*

**Model Summary**

| **Deviance R-Sq** | **Deviance R-Sq(adj)** | **AIC** | **AICc** | **BIC** | **Area Under ROC Curve** |
| --- | --- | --- | --- | --- | --- |
| 46.16% | 34.50% | 28.46 | 31.62 | 34.56 | 0.8896 |

**Goodness-of-Fit Tests**

| **Test** | **DF** | **Chi-Square** | **P-Value** |
| --- | --- | --- | --- |
| Deviance | 20 | 18.46 | 0.557 |
| Pearson | 20 | 15.00 | 0.776 |
| Hosmer-Lemeshow | 3 | 0.00 | 1.000 |

**Analysis of Variance**

|  |  | **Wald Test** | |
| --- | --- | --- | --- |
| **Source** | **DF** | **Chi-Square** | **P-Value** |
| Regression | 4 | 1.55 | 0.817 |
| group no. | 4 | 1.55 | 0.817 |

**Fits and Diagnostics for Unusual Observations**

| **Obs** | **Observed Probability** | **Fit** | **Resid** | **Std Resid** |  |
| --- | --- | --- | --- | --- | --- |
| 23 | 0.000 | 0.800 | -1.794 | -2.01 | R |

*R  Large residual*

MICE LETHALITY

**Binary Logistic Regression: Response of mice versus group no.**

* WARNING * The model could not be fit properly. Maximum likelihood estimates of parameters
may not exist due to quasi-complete separation of data points. The results might not be
reliable. Please refer to help for more information about quasi-complete separation.

**Method**

| Link function | Logit |
| --- | --- |
| Categorical predictor coding | (1, 0) |
| Rows used | 25 |

**Response Information**

| **Variable** | **Value** | **Count** |  |
| --- | --- | --- | --- |
| Response of mice | dead | 14 | (Event) |
|  | alive | 11 |  |
|  | Total | 25 |  |

**Regression Equation**

| P(dead) | | | = | exp(Y')/(1 + exp(Y')) |  |
| --- | --- | --- | --- | --- | --- |
| Y' | = | -0.405 + 1.79 group no._MXF 15 mg/kg + 0.0 group no._MXF 3.75 mg/kg + 0.81 group no._MXF 7.5 mg/kg - 13 group no._Negative Control + 14 group no._Positive Control | | | |

**Coefficients**

| **Term** | **Coef** | **SE Coef** | **Z-Value** | **P-Value** | **VIF** |
| --- | --- | --- | --- | --- | --- |
| Constant | -0.405 | 0.913 | -0.44 | 0.657 |  |
| group no. |  |  |  |  |  |
| MXF 15 mg/kg | 1.79 | 1.44 | 1.24 | 0.214 | 1.25 |
| MXF 7.5 mg/kg | 0.81 | 1.29 | 0.63 | 0.530 | 1.25 |
| Negative Control | -13 | 239 | -0.05 | 0.956 | 1.00 |
| Positive Control | 14 | 239 | 0.06 | 0.953 | 1.00 |

**Odds Ratios for Categorical Predictors**

| **Level A** | **Level B** | **Odds Ratio** | **95% CI** |
| --- | --- | --- | --- |
| group no. |  |  |  |
| MXF 15 mg/kg | MXF 3.75 mg/kg | 6.0000 | (0.3544, 101.5675) |
| MXF 7.5 mg/kg | MXF 3.75 mg/kg | 2.2500 | (0.1792, 28.2539) |
| Negative Control | MXF 3.75 mg/kg | 0.0000 | (0.0000, 1.25976E+198) |
| Positive Control | MXF 3.75 mg/kg | 1.16885E+06 | (0.0000, 7.64931E+209) |
| MXF 7.5 mg/kg | MXF 15 mg/kg | 0.3750 | (0.0222, 6.3480) |
| Negative Control | MXF 15 mg/kg | 0.0000 | (0.0000, 2.10318E+197) |
| Positive Control | MXF 15 mg/kg | 194808.4666 | (0.0000, 1.27706E+209) |
| Negative Control | MXF 7.5 mg/kg | 0.0000 | (0.0000, 5.59892E+197) |
| Positive Control | MXF 7.5 mg/kg | 519489.2443 | (0.0000, 3.39969E+209) |
| Positive Control | Negative Control | 6.07205E+11 | (0.0000, 1.04809E+300) |

*Odds ratio for level A relative to level B*

**Model Summary**

| **Deviance R-Sq** | **Deviance R-Sq(adj)** | **AIC** | **AICc** | **BIC** | **Area Under ROC Curve** |
| --- | --- | --- | --- | --- | --- |
| 46.16% | 34.50% | 28.46 | 31.62 | 34.56 | 0.8896 |

**Goodness-of-Fit Tests**

| **Test** | **DF** | **Chi-Square** | **P-Value** |
| --- | --- | --- | --- |
| Deviance | 20 | 18.46 | 0.557 |
| Pearson | 20 | 15.00 | 0.776 |
| Hosmer-Lemeshow | 3 | 0.00 | 1.000 |

**Analysis of Variance**

|  |  | **Wald Test** | |
| --- | --- | --- | --- |
| **Source** | **DF** | **Chi-Square** | **P-Value** |
| Regression | 4 | 1.55 | 0.817 |
| group no. | 4 | 1.55 | 0.817 |

**Fits and Diagnostics for Unusual Observations**

| **Obs** | **Observed Probability** | **Fit** | **Resid** | **Std Resid** |  |
| --- | --- | --- | --- | --- | --- |
| 23 | 0.000 | 0.800 | -1.794 | -2.01 | R |

*R  Large residual*

HA

**One-way ANOVA: Log2 HA Titer versus Group**

**Method**

| Null hypothesis | All means are equal |
| --- | --- |
| Alternative hypothesis | Not all means are equal |
| Significance level | α = 0.05 |

*Equal variances were assumed for the analysis.*

**Factor Information**

| **Factor** | **Levels** | **Values** |
| --- | --- | --- |
| Group | 5 | MXF 15mg/kg, MXF 3.5mg/kg, MXF 7.5mg/kg, Negative Control, Positive Control |

**Analysis of Variance**

| **Source** | **DF** | **Seq SS** | **Contribution** | **Adj SS** | **Adj MS** | **F-Value** | **P-Value** |
| --- | --- | --- | --- | --- | --- | --- | --- |
| Group | 4 | 59.84 | 78.49% | 59.84 | 14.9600 | 18.24 | 0.000 |
| Error | 20 | 16.40 | 21.51% | 16.40 | 0.8200 |  |  |
| Total | 24 | 76.24 | 100.00% |  |  |  |  |

**Model Summary**

| **S** | **R-sq** | **R-sq(adj)** | **PRESS** | **R-sq(pred)** |
| --- | --- | --- | --- | --- |
| 0.905539 | 78.49% | 74.19% | 25.625 | 66.39% |

**Means**

| **Group** | **N** | **Mean** | **StDev** | **95% CI** |
| --- | --- | --- | --- | --- |
| MXF 15mg/kg | 5 | 5.200 | 0.837 | (4.355, 6.045) |
| MXF 3.5mg/kg | 5 | 7.200 | 0.837 | (6.355, 8.045) |
| MXF 7.5mg/kg | 5 | 5.400 | 1.140 | (4.555, 6.245) |
| Negative Control | 5 | 6.800 | 0.837 | (5.955, 7.645) |
| Positive Control | 5 | 2.800 | 0.837 | (1.955, 3.645) |

*Pooled StDev = 0.905539*

**Tukey Pairwise Comparisons**

**Grouping Information Using the Tukey Method and 95% Confidence**

| **Group** | **N** | **Mean** | **Grouping** | | |
| --- | --- | --- | --- | --- | --- |
| MXF 3.5mg/kg | 5 | 7.200 | A |  |  |
| Negative Control | 5 | 6.800 | A | B |  |
| MXF 7.5mg/kg | 5 | 5.400 |  | B |  |
| MXF 15mg/kg | 5 | 5.200 |  | B |  |
| Positive Control | 5 | 2.800 |  |  | C |

*Means that do not share a letter are significantly different.*


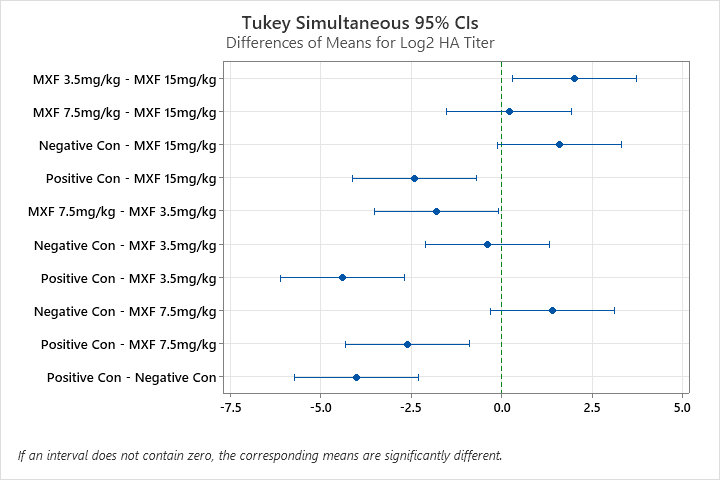


**Fisher Pairwise Comparisons**

**Grouping Information Using the Fisher LSD Method and 95% Confidence**

| **Group** | **N** | **Mean** | **Grouping** | | |
| --- | --- | --- | --- | --- | --- |
| MXF 3.5mg/kg | 5 | 7.200 | A |  |  |
| Negative Control | 5 | 6.800 | A |  |  |
| MXF 7.5mg/kg | 5 | 5.400 |  | B |  |
| MXF 15mg/kg | 5 | 5.200 |  | B |  |
| Positive Control | 5 | 2.800 |  |  | C |

*Means that do not share a letter are significantly different.*


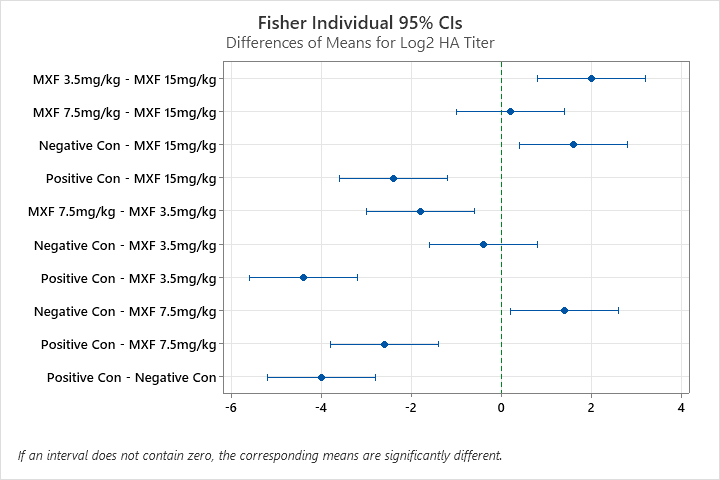


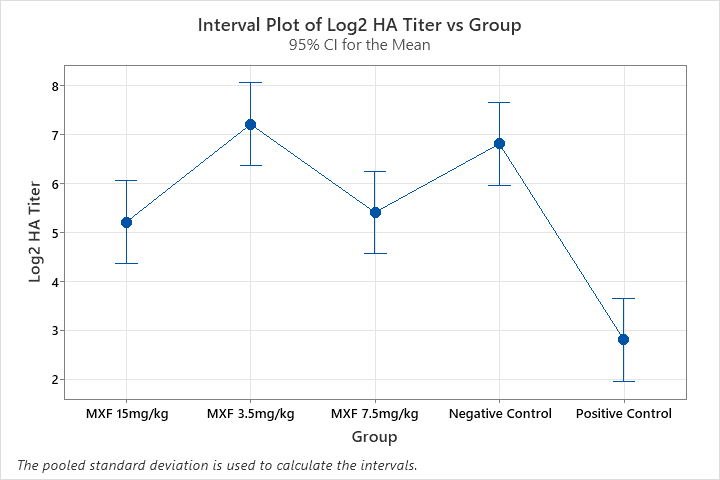


IN-VITRO

**One-way ANOVA: TGF-β (pg.ml) versus Group- in-vitro**

**Method**

| Null hypothesis | All means are equal |
| --- | --- |
| Alternative hypothesis | Not all means are equal |
| Significance level | α = 0.05 |

*Equal variances were assumed for the analysis.*

**Factor Information**

| **Factor** | **Levels** | **Values** |
| --- | --- | --- |
| Group | 6 | control, MXF 16µg/ml, MXF 32µg/ml, MXF 4µg/ml, MXF 64µg/ml, MXF 8µg/ml |

**Analysis of Variance**

| **Source** | **DF** | **Seq SS** | **Contribution** | **Adj SS** | **Adj MS** | **F-Value** | **P-Value** |
| --- | --- | --- | --- | --- | --- | --- | --- |
| Group | 5 | 141.77 | 78.04% | 141.77 | 28.354 | 8.53 | 0.001 |
| Error | 12 | 39.89 | 21.96% | 39.89 | 3.324 |  |  |
| Total | 17 | 181.66 | 100.00% |  |  |  |  |

**Model Summary**

| **S** | **R-sq** | **R-sq(adj)** | **PRESS** | **R-sq(pred)** |
| --- | --- | --- | --- | --- |
| 1.82322 | 78.04% | 68.89% | 89.7514 | 50.59% |

**Means**

| **Group** | **N** | **Mean** | **StDev** | **95% CI** |
| --- | --- | --- | --- | --- |
| control | 3 | 24.129 | 1.713 | (21.836, 26.423) |
| MXF 16µg/ml | 3 | 28.04 | 1.83 | (25.75, 30.34) |
| MXF 32µg/ml | 3 | 30.826 | 1.415 | (28.533, 33.120) |
| MXF 4µg/ml | 3 | 23.319 | 1.050 | (21.026, 25.613) |
| MXF 64µg/ml | 3 | 22.965 | 1.406 | (20.672, 25.259) |
| MXF 8µg/ml | 3 | 25.78 | 2.93 | (23.49, 28.07) |

*Pooled StDev = 1.82322*

**Tukey Pairwise Comparisons**

**Grouping Information Using the Tukey Method and 95% Confidence**

| **Group** | **N** | **Mean** | **Grouping** | | |
| --- | --- | --- | --- | --- | --- |
| MXF 32µg/ml | 3 | 30.826 | A |  |  |
| MXF 16µg/ml | 3 | 28.04 | A | B |  |
| MXF 8µg/ml | 3 | 25.78 |  | B | C |
| control | 3 | 24.129 |  | B | C |
| MXF 4µg/ml | 3 | 23.319 |  | B | C |
| MXF 64µg/ml | 3 | 22.965 |  |  | C |

*Means that do not share a letter are significantly different.*


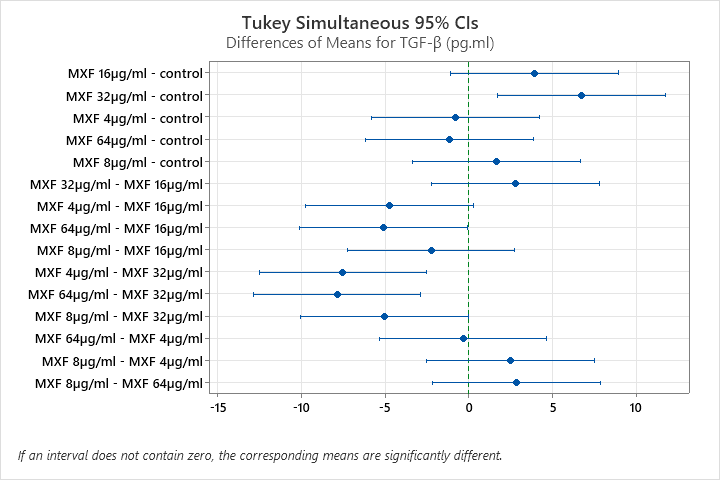


**Fisher Pairwise Comparisons**

**Grouping Information Using the Fisher LSD Method and 95% Confidence**

| **Group** | **N** | **Mean** | **Grouping** | | |
| --- | --- | --- | --- | --- | --- |
| MXF 32µg/ml | 3 | 30.826 | A |  |  |
| MXF 16µg/ml | 3 | 28.04 | A | B |  |
| MXF 8µg/ml | 3 | 25.78 |  | B | C |
| control | 3 | 24.129 |  |  | C |
| MXF 4µg/ml | 3 | 23.319 |  |  | C |
| MXF 64µg/ml | 3 | 22.965 |  |  | C |

*Means that do not share a letter are significantly different.*


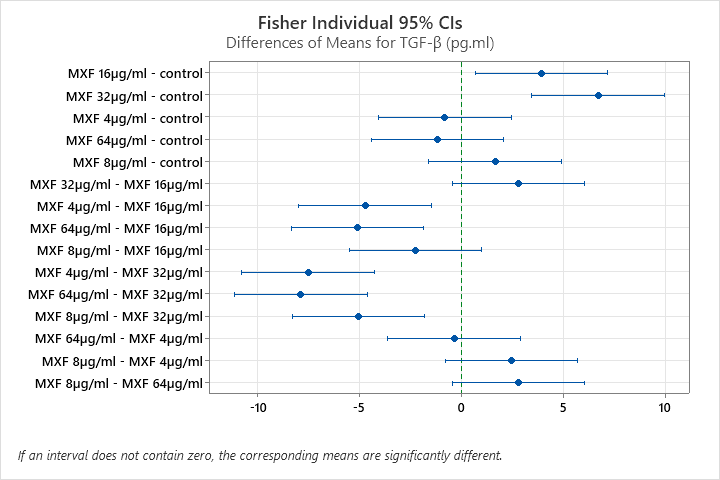


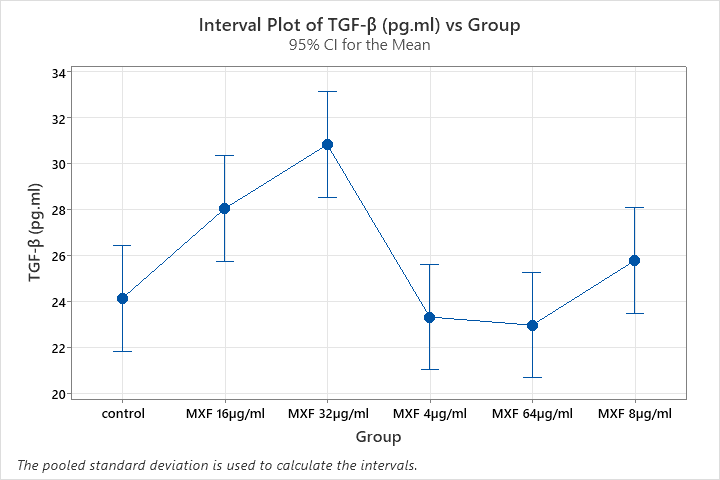


IN-VITRO

**One-way ANOVA: IL-10 (pg.ml) versus Group**

**Method**

| Null hypothesis | All means are equal |
| --- | --- |
| Alternative hypothesis | Not all means are equal |
| Significance level | α = 0.05 |

*Equal variances were assumed for the analysis.*

**Factor Information**

| **Factor** | **Levels** | **Values** |
| --- | --- | --- |
| Group | 6 | control, MXF 16µg/ml, MXF 32µg/ml, MXF 4µg/ml, MXF 64µg/ml, MXF 8µg/ml |

**Analysis of Variance**

| **Source** | **DF** | **Seq SS** | **Contribution** | **Adj SS** | **Adj MS** | **F-Value** | **P-Value** |
| --- | --- | --- | --- | --- | --- | --- | --- |
| Group | 5 | 2552.00 | 99.04% | 2552.00 | 510.401 | 247.75 | 0.000 |
| Error | 12 | 24.72 | 0.96% | 24.72 | 2.060 |  |  |
| Total | 17 | 2576.72 | 100.00% |  |  |  |  |

**Model Summary**

| **S** | **R-sq** | **R-sq(adj)** | **PRESS** | **R-sq(pred)** |
| --- | --- | --- | --- | --- |
| 1.43532 | 99.04% | 98.64% | 55.6239 | 97.84% |

**Means**

| **Group** | **N** | **Mean** | **StDev** | **95% CI** |
| --- | --- | --- | --- | --- |
| control | 3 | 20.325 | 1.304 | (18.520, 22.131) |
| MXF 16µg/ml | 3 | 50.427 | 1.362 | (48.621, 52.232) |
| MXF 32µg/ml | 3 | 36.417 | 1.627 | (34.612, 38.223) |
| MXF 4µg/ml | 3 | 18.83 | 1.74 | (17.02, 20.63) |
| MXF 64µg/ml | 3 | 18.585 | 1.409 | (16.780, 20.391) |
| MXF 8µg/ml | 3 | 21.219 | 1.069 | (19.413, 23.024) |

*Pooled StDev = 1.43532*

**Tukey Pairwise Comparisons**

**Grouping Information Using the Tukey Method and 95% Confidence**

| **Group** | **N** | **Mean** | **Grouping** | | |
| --- | --- | --- | --- | --- | --- |
| MXF 16µg/ml | 3 | 50.427 | A |  |  |
| MXF 32µg/ml | 3 | 36.417 |  | B |  |
| MXF 8µg/ml | 3 | 21.219 |  |  | C |
| control | 3 | 20.325 |  |  | C |
| MXF 4µg/ml | 3 | 18.83 |  |  | C |
| MXF 64µg/ml | 3 | 18.585 |  |  | C |

*Means that do not share a letter are significantly different.*


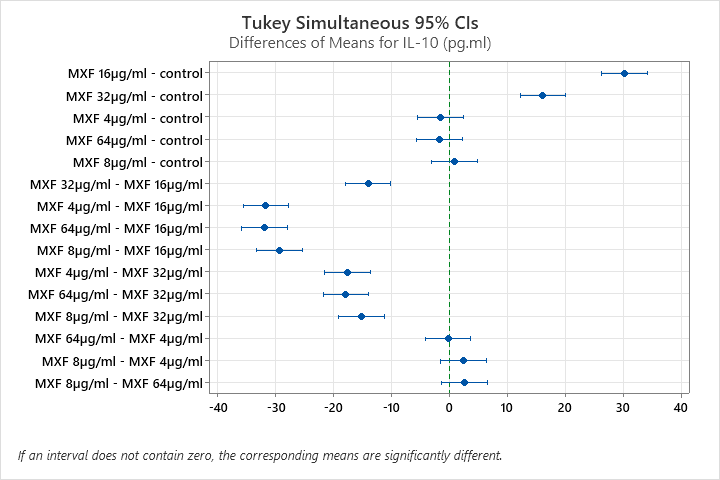


**Fisher Pairwise Comparisons**

**Grouping Information Using the Fisher LSD Method and 95% Confidence**

| **Group** | **N** | **Mean** | **Grouping** | | | |
| --- | --- | --- | --- | --- | --- | --- |
| MXF 16µg/ml | 3 | 50.427 | A |  |  |  |
| MXF 32µg/ml | 3 | 36.417 |  | B |  |  |
| MXF 8µg/ml | 3 | 21.219 |  |  | C |  |
| control | 3 | 20.325 |  |  | C | D |
| MXF 4µg/ml | 3 | 18.83 |  |  | C | D |
| MXF 64µg/ml | 3 | 18.585 |  |  |  | D |

*Means that do not share a letter are significantly different.*


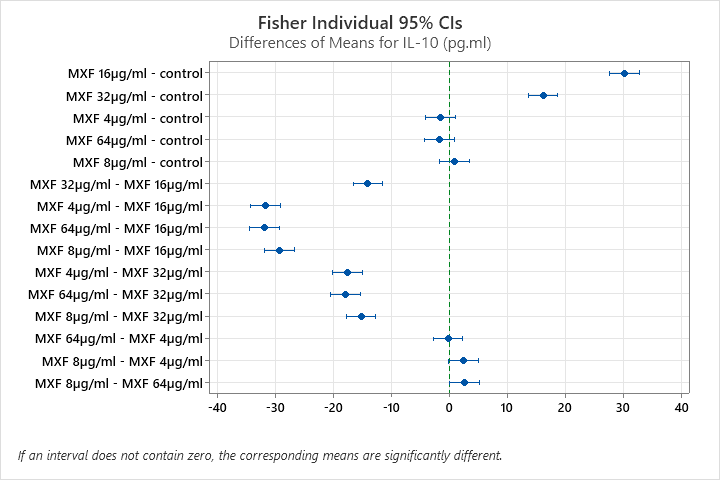


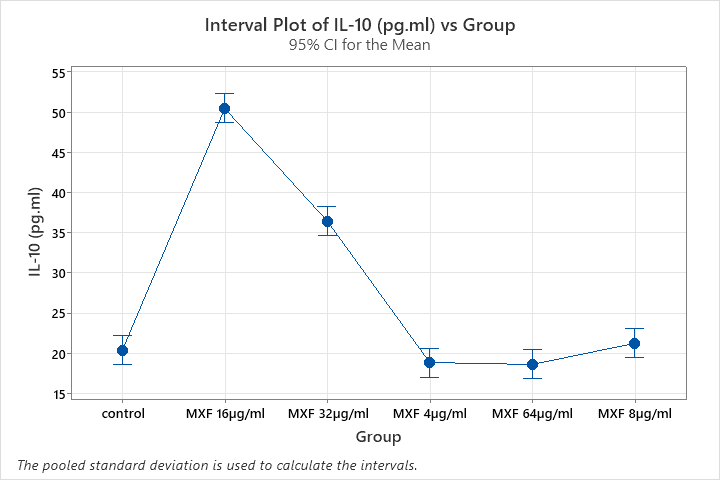


IN-VITRO

**One-way ANOVA: IL-6 (pg.ml) versus Group**

**Method**

| Null hypothesis | All means are equal |
| --- | --- |
| Alternative hypothesis | Not all means are equal |
| Significance level | α = 0.05 |

*Equal variances were assumed for the analysis.*

**Factor Information**

| **Factor** | **Levels** | **Values** |
| --- | --- | --- |
| Group | 6 | control, MXF 16µg/ml, MXF 32µg/ml, MXF 4µg/ml, MXF 64µg/ml, MXF 8µg/ml |

**Analysis of Variance**

| **Source** | **DF** | **Seq SS** | **Contribution** | **Adj SS** | **Adj MS** | **F-Value** | **P-Value** |
| --- | --- | --- | --- | --- | --- | --- | --- |
| Group | 5 | 63.48 | 74.34% | 63.48 | 12.697 | 6.95 | 0.003 |
| Error | 12 | 21.91 | 25.66% | 21.91 | 1.826 |  |  |
| Total | 17 | 85.39 | 100.00% |  |  |  |  |

**Model Summary**

| **S** | **R-sq** | **R-sq(adj)** | **PRESS** | **R-sq(pred)** |
| --- | --- | --- | --- | --- |
| 1.35114 | 74.34% | 63.66% | 49.2909 | 42.28% |

**Means**

| **Group** | **N** | **Mean** | **StDev** | **95% CI** |
| --- | --- | --- | --- | --- |
| control | 3 | 13.805 | 0.714 | (12.105, 15.504) |
| MXF 16µg/ml | 3 | 11.644 | 0.865 | (9.944, 13.343) |
| MXF 32µg/ml | 3 | 12.20 | 2.03 | (10.50, 13.90) |
| MXF 4µg/ml | 3 | 14.57 | 1.95 | (12.87, 16.27) |
| MXF 64µg/ml | 3 | 8.714 | 1.121 | (7.015, 10.414) |
| MXF 8µg/ml | 3 | 11.390 | 0.717 | (9.691, 13.090) |

*Pooled StDev = 1.35114*

**Tukey Pairwise Comparisons**

**Grouping Information Using the Tukey Method and 95% Confidence**

| **Group** | **N** | **Mean** | **Grouping** | |
| --- | --- | --- | --- | --- |
| MXF 4µg/ml | 3 | 14.57 | A |  |
| control | 3 | 13.805 | A |  |
| MXF 32µg/ml | 3 | 12.20 | A | B |
| MXF 16µg/ml | 3 | 11.644 | A | B |
| MXF 8µg/ml | 3 | 11.390 | A | B |
| MXF 64µg/ml | 3 | 8.714 |  | B |

*Means that do not share a letter are significantly different.*


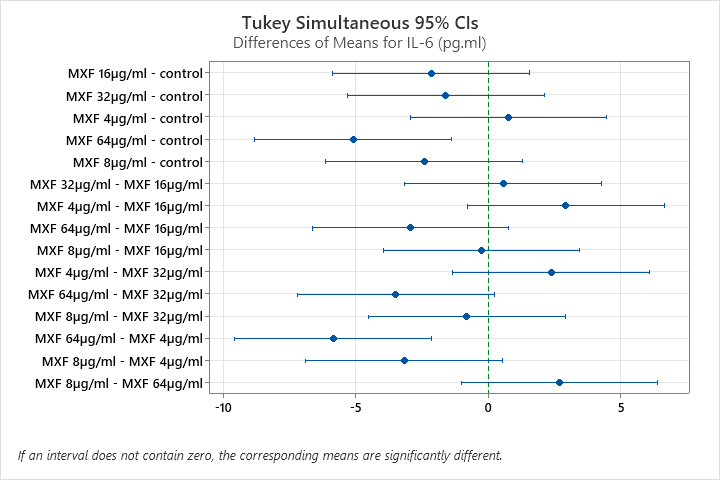


**Fisher Pairwise Comparisons**

**Grouping Information Using the Fisher LSD Method and 95% Confidence**

| **Group** | **N** | **Mean** | **Grouping** | | | |
| --- | --- | --- | --- | --- | --- | --- |
| MXF 4µg/ml | 3 | 14.57 | A |  |  |  |
| control | 3 | 13.805 | A | B |  |  |
| MXF 32µg/ml | 3 | 12.20 | A | B | C |  |
| MXF 16µg/ml | 3 | 11.644 |  | B | C |  |
| MXF 8µg/ml | 3 | 11.390 |  |  | C |  |
| MXF 64µg/ml | 3 | 8.714 |  |  |  | D |

*Means that do not share a letter are significantly different.*


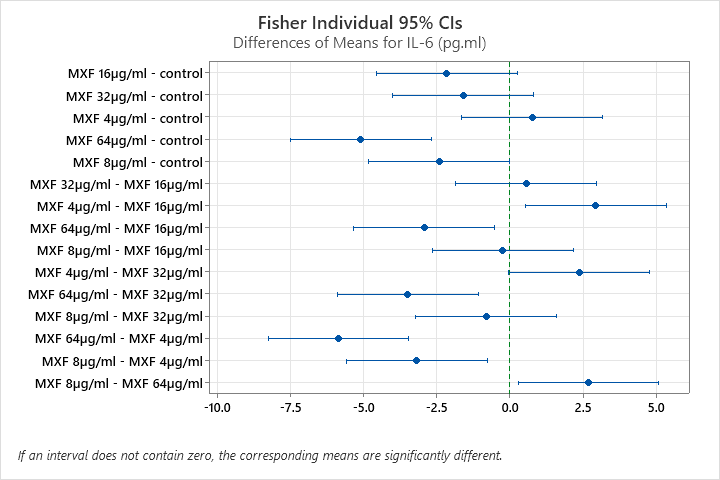


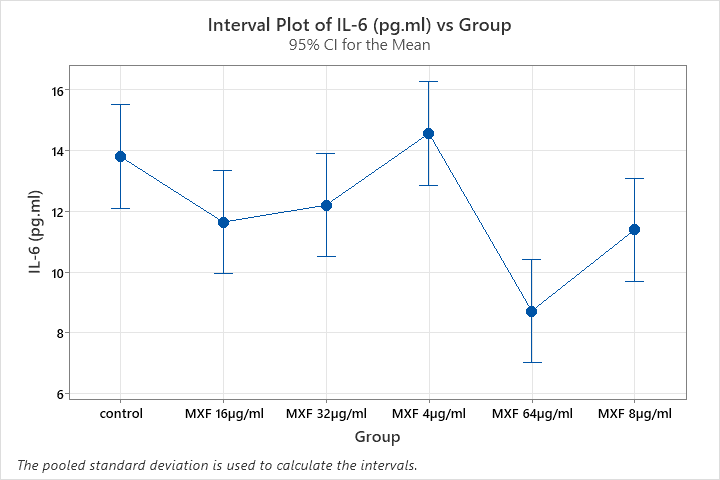


IN-VITRO

**One-way ANOVA: TNF-α (pg.ml) versus Group**

**Method**

| Null hypothesis | All means are equal |
| --- | --- |
| Alternative hypothesis | Not all means are equal |
| Significance level | α = 0.05 |

*Equal variances were assumed for the analysis.*

**Factor Information**

| **Factor** | **Levels** | **Values** |
| --- | --- | --- |
| Group | 6 | control, MXF 16µg/ml, MXF 32µg/ml, MXF 4µg/ml, MXF 64µg/ml, MXF 8µg/ml |

**Analysis of Variance**

| **Source** | **DF** | **Seq SS** | **Contribution** | **Adj SS** | **Adj MS** | **F-Value** | **P-Value** |
| --- | --- | --- | --- | --- | --- | --- | --- |
| Group | 5 | 279.5 | 63.42% | 279.5 | 55.89 | 4.16 | 0.020 |
| Error | 12 | 161.2 | 36.58% | 161.2 | 13.43 |  |  |
| Total | 17 | 440.7 | 100.00% |  |  |  |  |

**Model Summary**

| **S** | **R-sq** | **R-sq(adj)** | **PRESS** | **R-sq(pred)** |
| --- | --- | --- | --- | --- |
| 3.66507 | 63.42% | 48.18% | 362.685 | 17.70% |

**Means**

| **Group** | **N** | **Mean** | **StDev** | **95% CI** |
| --- | --- | --- | --- | --- |
| control | 3 | 41.40 | 4.43 | (36.78, 46.01) |
| MXF 16µg/ml | 3 | 36.32 | 3.32 | (31.71, 40.93) |
| MXF 32µg/ml | 3 | 37.53 | 2.25 | (32.92, 42.14) |
| MXF 4µg/ml | 3 | 39.51 | 2.95 | (34.90, 44.12) |
| MXF 64µg/ml | 3 | 28.81 | 5.62 | (24.20, 33.43) |
| MXF 8µg/ml | 3 | 36.24 | 2.15 | (31.63, 40.85) |

*Pooled StDev = 3.66507*

**Tukey Pairwise Comparisons**

**Grouping Information Using the Tukey Method and 95% Confidence**

| **Group** | **N** | **Mean** | **Grouping** | |
| --- | --- | --- | --- | --- |
| control | 3 | 41.40 | A |  |
| MXF 4µg/ml | 3 | 39.51 | A |  |
| MXF 32µg/ml | 3 | 37.53 | A | B |
| MXF 16µg/ml | 3 | 36.32 | A | B |
| MXF 8µg/ml | 3 | 36.24 | A | B |
| MXF 64µg/ml | 3 | 28.81 |  | B |

*Means that do not share a letter are significantly different.*


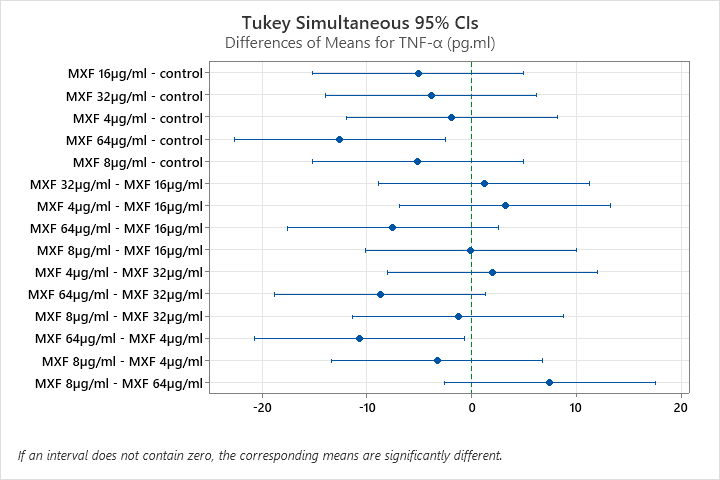


**Fisher Pairwise Comparisons**

**Grouping Information Using the Fisher LSD Method and 95% Confidence**

| **Group** | **N** | **Mean** | **Grouping** | |
| --- | --- | --- | --- | --- |
| control | 3 | 41.40 | A |  |
| MXF 4µg/ml | 3 | 39.51 | A |  |
| MXF 32µg/ml | 3 | 37.53 | A |  |
| MXF 16µg/ml | 3 | 36.32 | A |  |
| MXF 8µg/ml | 3 | 36.24 | A |  |
| MXF 64µg/ml | 3 | 28.81 |  | B |

*Means that do not share a letter are significantly different.*


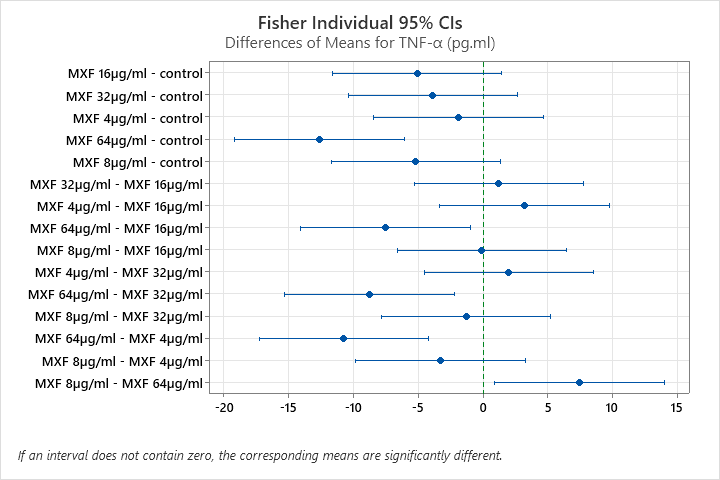


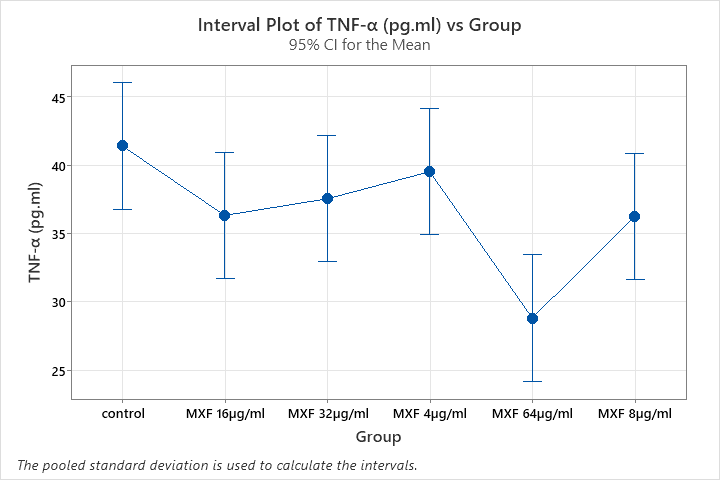


IN-VIVO

**One-way ANOVA: TGF-β (pg.ml) versus Group- in-vivo**

**Method**

| Null hypothesis | All means are equal |
| --- | --- |
| Alternative hypothesis | Not all means are equal |
| Significance level | α = 0.05 |

*Equal variances were assumed for the analysis.*

**Factor Information**

| **Factor** | **Levels** | **Values** |
| --- | --- | --- |
| Group | 5 | MXF 15mg/kg, MXF 3.75mg/kg, MXF 7.5mg/kg, Negative Control, Positive Control |

**Analysis of Variance**

| **Source** | **DF** | **Seq SS** | **Contribution** | **Adj SS** | **Adj MS** | **F-Value** | **P-Value** |
| --- | --- | --- | --- | --- | --- | --- | --- |
| Group | 4 | 97619 | 68.66% | 97619 | 24405 | 8.21 | 0.001 |
| Error | 15 | 44562 | 31.34% | 44562 | 2971 |  |  |
| Total | 19 | 142181 | 100.00% |  |  |  |  |

**Model Summary**

| **S** | **R-sq** | **R-sq(adj)** | **PRESS** | **R-sq(pred)** |
| --- | --- | --- | --- | --- |
| 54.5052 | 68.66% | 60.30% | 79221.9 | 44.28% |

**Means**

| **Group** | **N** | **Mean** | **StDev** | **95% CI** |
| --- | --- | --- | --- | --- |
| MXF 15mg/kg | 4 | 403.1 | 61.2 | (345.1, 461.2) |
| MXF 3.75mg/kg | 4 | 232.0 | 41.6 | (173.9, 290.1) |
| MXF 7.5mg/kg | 4 | 358.1 | 55.9 | (300.0, 416.2) |
| Negative Control | 4 | 253.3 | 28.4 | (195.2, 311.4) |
| Positive Control | 4 | 383.5 | 73.8 | (325.4, 441.6) |

*Pooled StDev = 54.5052*

**Tukey Pairwise Comparisons**

**Grouping Information Using the Tukey Method and 95% Confidence**

| **Group** | **N** | **Mean** | **Grouping** | | |
| --- | --- | --- | --- | --- | --- |
| MXF 15mg/kg | 4 | 403.1 | A |  |  |
| Positive Control | 4 | 383.5 | A |  |  |
| MXF 7.5mg/kg | 4 | 358.1 | A | B |  |
| Negative Control | 4 | 253.3 |  | B | C |
| MXF 3.75mg/kg | 4 | 232.0 |  |  | C |

*Means that do not share a letter are significantly different.*


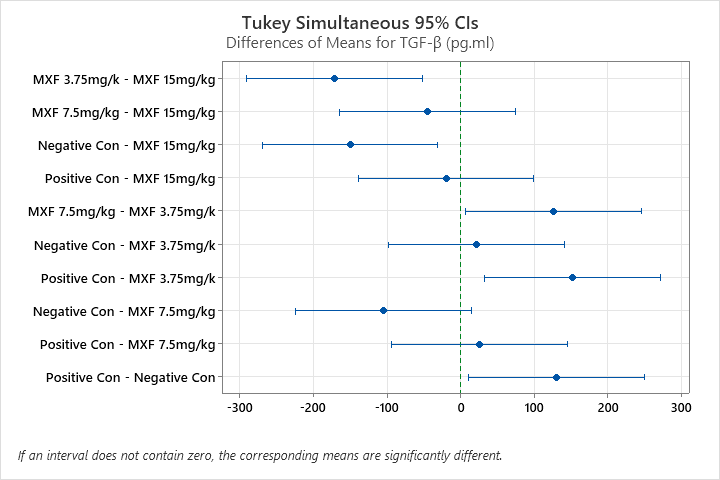


**Fisher Pairwise Comparisons**

**Grouping Information Using the Fisher LSD Method and 95% Confidence**

| **Group** | **N** | **Mean** | **Grouping** | |
| --- | --- | --- | --- | --- |
| MXF 15mg/kg | 4 | 403.1 | A |  |
| Positive Control | 4 | 383.5 | A |  |
| MXF 7.5mg/kg | 4 | 358.1 | A |  |
| Negative Control | 4 | 253.3 |  | B |
| MXF 3.75mg/kg | 4 | 232.0 |  | B |

*Means that do not share a letter are significantly different.*


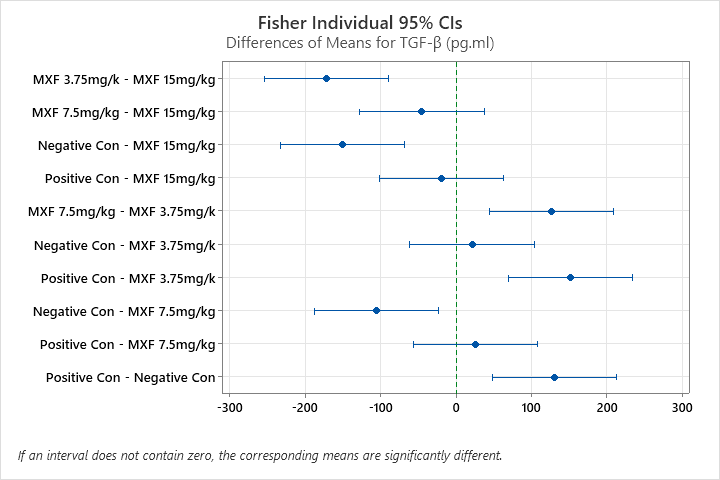


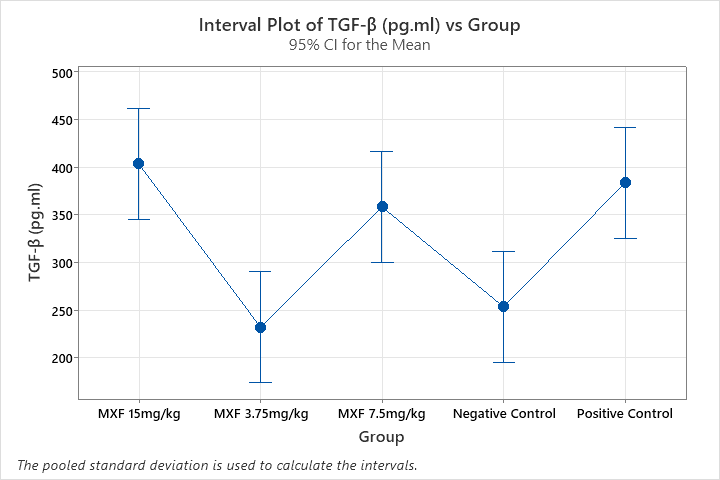


IN-VIVO

**One-way ANOVA: IL-10 (pg.ml) versus Group**

**Method**

| Null hypothesis | All means are equal |
| --- | --- |
| Alternative hypothesis | Not all means are equal |
| Significance level | α = 0.05 |

*Equal variances were assumed for the analysis.*

**Factor Information**

| **Factor** | **Levels** | **Values** |
| --- | --- | --- |
| Group | 5 | MXF 15mg/kg, MXF 3.75mg/kg, MXF 7.5mg/kg, Negative Control, Positive Control |

**Analysis of Variance**

| **Source** | **DF** | **Seq SS** | **Contribution** | **Adj SS** | **Adj MS** | **F-Value** | **P-Value** |
| --- | --- | --- | --- | --- | --- | --- | --- |
| Group | 4 | 2918.6 | 78.10% | 2918.6 | 729.65 | 13.37 | 0.000 |
| Error | 15 | 818.6 | 21.90% | 818.6 | 54.57 |  |  |
| Total | 19 | 3737.2 | 100.00% |  |  |  |  |

**Model Summary**

| **S** | **R-sq** | **R-sq(adj)** | **PRESS** | **R-sq(pred)** |
| --- | --- | --- | --- | --- |
| 7.38729 | 78.10% | 72.26% | 1455.26 | 61.06% |

**Means**

| **Group** | **N** | **Mean** | **StDev** | **95% CI** |
| --- | --- | --- | --- | --- |
| MXF 15mg/kg | 4 | 17.57 | 0.00 | (9.70, 25.44) |
| MXF 3.75mg/kg | 4 | 43.02 | 6.63 | (35.15, 50.90) |
| MXF 7.5mg/kg | 4 | 30.78 | 14.92 | (22.90, 38.65) |
| Negative Control | 4 | 20.345 | 1.580 | (12.472, 28.218) |
| Positive Control | 4 | 48.205 | 1.916 | (40.332, 56.078) |

*Pooled StDev = 7.38729*

**Tukey Pairwise Comparisons**

**Grouping Information Using the Tukey Method and 95% Confidence**

| **Group** | **N** | **Mean** | **Grouping** | | |
| --- | --- | --- | --- | --- | --- |
| Positive Control | 4 | 48.205 | A |  |  |
| MXF 3.75mg/kg | 4 | 43.02 | A | B |  |
| MXF 7.5mg/kg | 4 | 30.78 |  | B | C |
| Negative Control | 4 | 20.345 |  |  | C |
| MXF 15mg/kg | 4 | 17.57 |  |  | C |

*Means that do not share a letter are significantly different.*


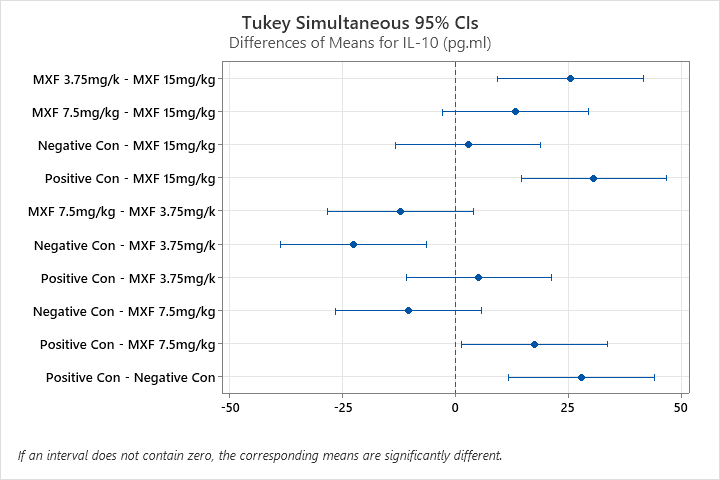


**Fisher Pairwise Comparisons**

**Grouping Information Using the Fisher LSD Method and 95% Confidence**

| **Group** | **N** | **Mean** | **Grouping** | | |
| --- | --- | --- | --- | --- | --- |
| Positive Control | 4 | 48.205 | A |  |  |
| MXF 3.75mg/kg | 4 | 43.02 | A |  |  |
| MXF 7.5mg/kg | 4 | 30.78 |  | B |  |
| Negative Control | 4 | 20.345 |  | B | C |
| MXF 15mg/kg | 4 | 17.57 |  |  | C |

*Means that do not share a letter are significantly different.*


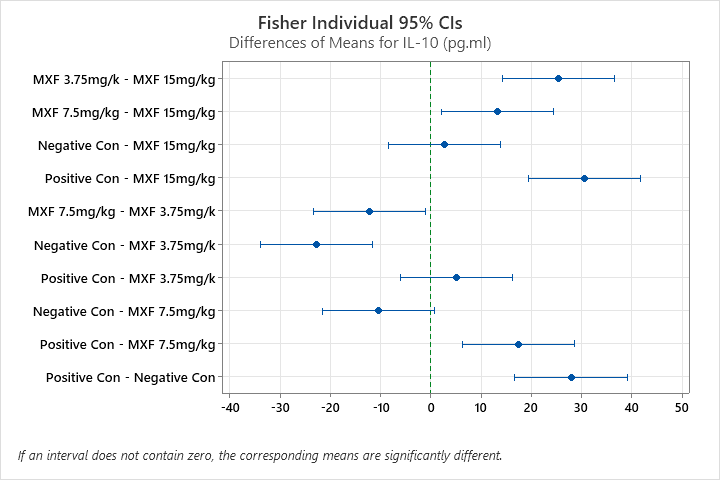


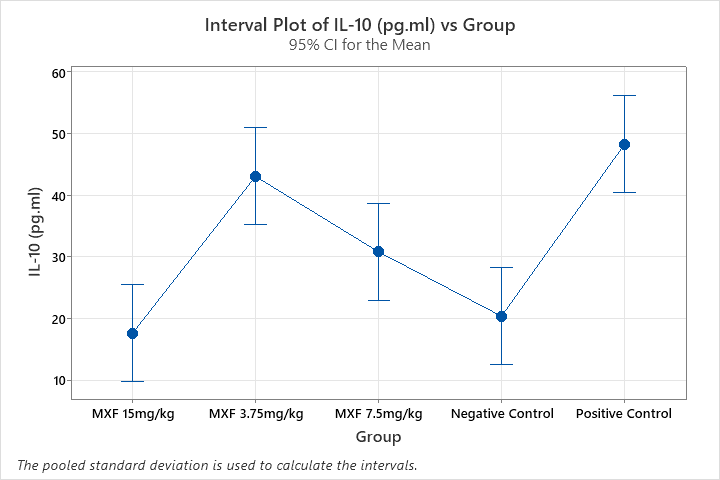


IN-VIVO

**One-way ANOVA: IL-6 (pg.ml) versus Group**

**Method**

| Null hypothesis | All means are equal |
| --- | --- |
| Alternative hypothesis | Not all means are equal |
| Significance level | α = 0.05 |

*Equal variances were assumed for the analysis.*

**Factor Information**

| **Factor** | **Levels** | **Values** |
| --- | --- | --- |
| Group | 5 | MXF 15mg/kg, MXF 3.75mg/kg, MXF 7.5mg/kg, Negative Control, Positive Control |

**Analysis of Variance**

| **Source** | **DF** | **Seq SS** | **Contribution** | **Adj SS** | **Adj MS** | **F-Value** | **P-Value** |
| --- | --- | --- | --- | --- | --- | --- | --- |
| Group | 4 | 118.66 | 78.14% | 118.66 | 29.665 | 13.41 | 0.000 |
| Error | 15 | 33.19 | 21.86% | 33.19 | 2.213 |  |  |
| Total | 19 | 151.85 | 100.00% |  |  |  |  |

**Model Summary**

| **S** | **R-sq** | **R-sq(adj)** | **PRESS** | **R-sq(pred)** |
| --- | --- | --- | --- | --- |
| 1.48760 | 78.14% | 72.31% | 59.0121 | 61.14% |

**Means**

| **Group** | **N** | **Mean** | **StDev** | **95% CI** |
| --- | --- | --- | --- | --- |
| MXF 15mg/kg | 4 | 8.570 | 0.000 | (6.985, 10.155) |
| MXF 3.75mg/kg | 4 | 13.783 | 0.781 | (12.197, 15.368) |
| MXF 7.5mg/kg | 4 | 11.21 | 2.66 | (9.63, 12.80) |
| Negative Control | 4 | 15.628 | 1.764 | (14.042, 17.213) |
| Positive Control | 4 | 13.572 | 0.531 | (11.987, 15.158) |

*Pooled StDev = 1.48760*

**Tukey Pairwise Comparisons**

**Grouping Information Using the Tukey Method and 95% Confidence**

| **Group** | **N** | **Mean** | **Grouping** | | |
| --- | --- | --- | --- | --- | --- |
| Negative Control | 4 | 15.628 | A |  |  |
| MXF 3.75mg/kg | 4 | 13.783 | A | B |  |
| Positive Control | 4 | 13.572 | A | B |  |
| MXF 7.5mg/kg | 4 | 11.21 |  | B | C |
| MXF 15mg/kg | 4 | 8.570 |  |  | C |

*Means that do not share a letter are significantly different.*


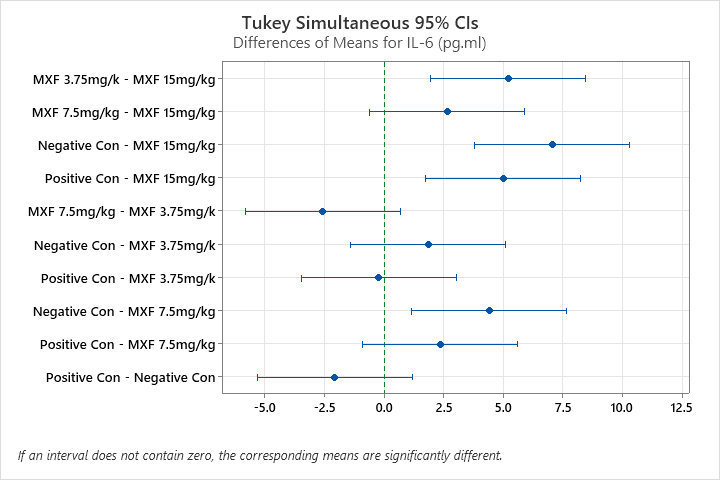


**Fisher Pairwise Comparisons**

**Grouping Information Using the Fisher LSD Method and 95% Confidence**

| **Group** | **N** | **Mean** | **Grouping** | | |
| --- | --- | --- | --- | --- | --- |
| Negative Control | 4 | 15.628 | A |  |  |
| MXF 3.75mg/kg | 4 | 13.783 | A |  |  |
| Positive Control | 4 | 13.572 | A |  |  |
| MXF 7.5mg/kg | 4 | 11.21 |  | B |  |
| MXF 15mg/kg | 4 | 8.570 |  |  | C |

*Means that do not share a letter are significantly different.*


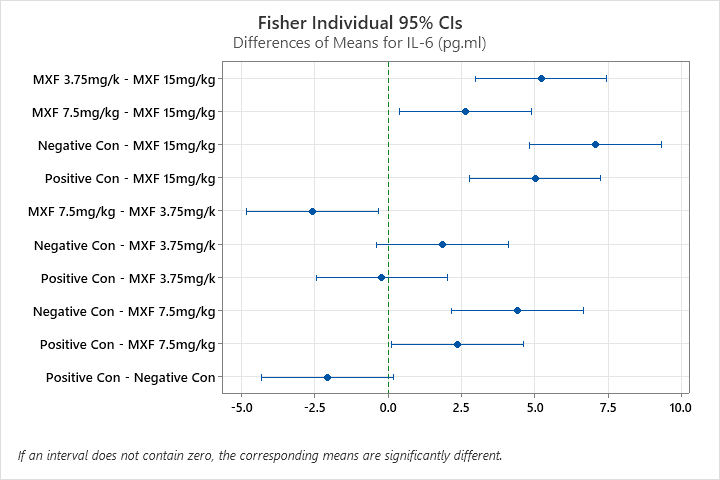


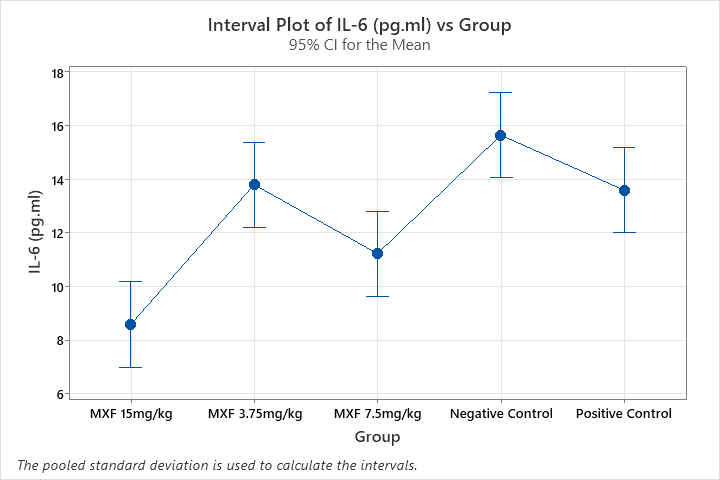


IN-VIVO

**One-way ANOVA: TNF-α (pg.ml) versus Group**

**Method**

| Null hypothesis | All means are equal |
| --- | --- |
| Alternative hypothesis | Not all means are equal |
| Significance level | α = 0.05 |

*Equal variances were assumed for the analysis.*

**Factor Information**

| **Factor** | **Levels** | **Values** |
| --- | --- | --- |
| Group | 5 | MXF 15mg/kg, MXF 3.75mg/kg, MXF 7.5mg/kg, Negative Control, Positive Control |

**Analysis of Variance**

| **Source** | **DF** | **Seq SS** | **Contribution** | **Adj SS** | **Adj MS** | **F-Value** | **P-Value** |
| --- | --- | --- | --- | --- | --- | --- | --- |
| Group | 4 | 876.4 | 87.11% | 876.4 | 219.097 | 25.34 | 0.000 |
| Error | 15 | 129.7 | 12.89% | 129.7 | 8.645 |  |  |
| Total | 19 | 1006.1 | 100.00% |  |  |  |  |

**Model Summary**

| **S** | **R-sq** | **R-sq(adj)** | **PRESS** | **R-sq(pred)** |
| --- | --- | --- | --- | --- |
| 2.94020 | 87.11% | 83.67% | 230.527 | 77.09% |

**Means**

| **Group** | **N** | **Mean** | **StDev** | **95% CI** |
| --- | --- | --- | --- | --- |
| MXF 15mg/kg | 4 | 20.99 | 0.00 | (17.86, 24.12) |
| MXF 3.75mg/kg | 4 | 35.49 | 2.18 | (32.36, 38.62) |
| MXF 7.5mg/kg | 4 | 33.84 | 3.40 | (30.71, 36.98) |
| Negative Control | 4 | 40.31 | 5.00 | (37.17, 43.44) |
| Positive Control | 4 | 36.990 | 1.379 | (33.857, 40.123) |

*Pooled StDev = 2.94020*

**Tukey Pairwise Comparisons**

**Grouping Information Using the Tukey Method and 95% Confidence**

| **Group** | **N** | **Mean** | **Grouping** | | |
| --- | --- | --- | --- | --- | --- |
| Negative Control | 4 | 40.31 | A |  |  |
| Positive Control | 4 | 36.990 | A | B |  |
| MXF 3.75mg/kg | 4 | 35.49 | A | B |  |
| MXF 7.5mg/kg | 4 | 33.84 |  | B |  |
| MXF 15mg/kg | 4 | 20.99 |  |  | C |

*Means that do not share a letter are significantly different.*


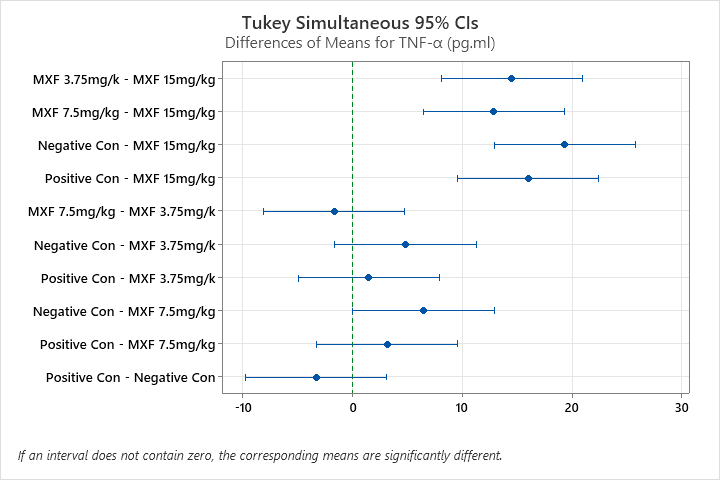


**Fisher Pairwise Comparisons**

**Grouping Information Using the Fisher LSD Method and 95% Confidence**

| **Group** | **N** | **Mean** | **Grouping** | | |
| --- | --- | --- | --- | --- | --- |
| Negative Control | 4 | 40.31 | A |  |  |
| Positive Control | 4 | 36.990 | A | B |  |
| MXF 3.75mg/kg | 4 | 35.49 |  | B |  |
| MXF 7.5mg/kg | 4 | 33.84 |  | B |  |
| MXF 15mg/kg | 4 | 20.99 |  |  | C |

*Means that do not share a letter are significantly different.*


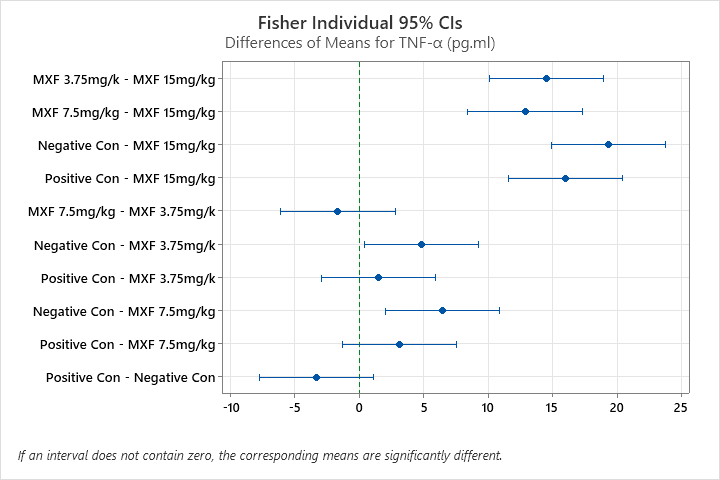


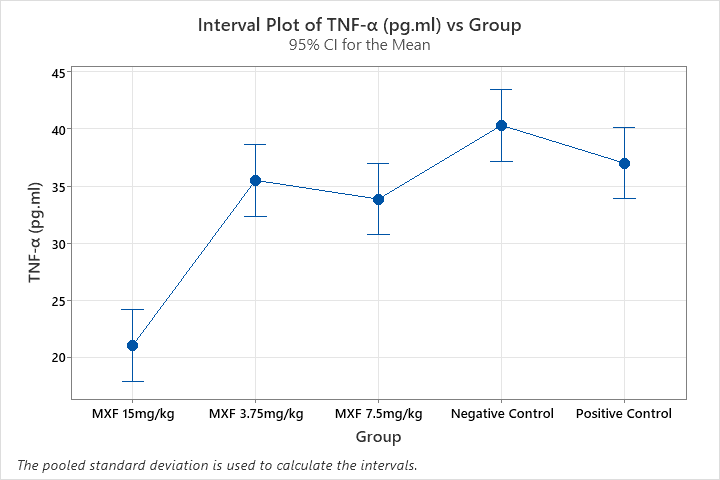


K AND ALPHA

**Descriptive Statistics: K (Carbon Clearance Rate)**

**Statistics**

| **Variable** | **Group** | **Mean** | **SE Mean** |
| --- | --- | --- | --- |
| K (Carbon Clearance Rate) | Control group | 0.022386 | 0.000985 |
|  | Moxifloxacin 15mg/kg | 0.01331 | 0.00310 |
|  | Moxifloxacin 3.75mg/kg | 0.02379 | 0.00207 |
|  | Moxifloxacin 7.5mg/kg | 0.01600 | 0.00175 |

K AND ALPHA

**Descriptive Statistics: α (Phagocytic index)**

**Statistics**

| **Variable** | **Group** | **Mean** | **SE Mean** |
| --- | --- | --- | --- |
| α (Phagocytic index) | Control group | 6.5251 | 0.0738 |
|  | Moxifloxacin 15mg/kg | 5.501 | 0.439 |
|  | Moxifloxacin 3.75mg/kg | 6.782 | 0.264 |
|  | Moxifloxacin 7.5mg/kg | 6.064 | 0.126 |

HA

**Descriptive Statistics: Log2 HA Titer**

**Statistics**

| **Variable** | **Group** | **Mean** | **SE Mean** |
| --- | --- | --- | --- |
| Log2 HA Titer | MXF 15mg/kg | 5.200 | 0.374 |
|  | MXF 3.5mg/kg | 7.200 | 0.374 |
|  | MXF 7.5mg/kg | 5.400 | 0.510 |
|  | Negative Control | 6.800 | 0.374 |
|  | Positive Control | 2.800 | 0.374 |

IN-VITRO

**Descriptive Statistics: TGF-β (pg.ml), IL-10 (pg.ml), IL-6 (pg.ml), TNF-α (pg.ml)**

**Statistics**

| **Variable** | **Group** | **Mean** | **SE Mean** |
| --- | --- | --- | --- |
| TGF-β (pg.ml) | control | 24.129 | 0.989 |
|  | MXF 16µg/ml | 28.04 | 1.05 |
|  | MXF 32µg/ml | 30.826 | 0.817 |
|  | MXF 4µg/ml | 23.319 | 0.606 |
|  | MXF 64µg/ml | 22.965 | 0.812 |
|  | MXF 8µg/ml | 25.78 | 1.69 |
|  |  |  |  |
| IL-10 (pg.ml) | control | 20.325 | 0.753 |
|  | MXF 16µg/ml | 50.427 | 0.786 |
|  | MXF 32µg/ml | 36.417 | 0.940 |
|  | MXF 4µg/ml | 18.83 | 1.01 |
|  | MXF 64µg/ml | 18.585 | 0.814 |
|  | MXF 8µg/ml | 21.219 | 0.617 |
|  |  |  |  |
| IL-6 (pg.ml) | control | 13.805 | 0.412 |
|  | MXF 16µg/ml | 11.644 | 0.499 |
|  | MXF 32µg/ml | 12.20 | 1.17 |
|  | MXF 4µg/ml | 14.57 | 1.13 |
|  | MXF 64µg/ml | 8.714 | 0.647 |
|  | MXF 8µg/ml | 11.390 | 0.414 |
|  |  |  |  |
| TNF-α (pg.ml) | control | 41.40 | 2.56 |
|  | MXF 16µg/ml | 36.32 | 1.92 |
|  | MXF 32µg/ml | 37.53 | 1.30 |
|  | MXF 4µg/ml | 39.51 | 1.70 |
|  | MXF 64µg/ml | 28.81 | 3.24 |
|  | MXF 8µg/ml | 36.24 | 1.24 |

IN-VIVO

**Descriptive Statistics: TGF-β (pg.ml), IL-10 (pg.ml), IL-6 (pg.ml), TNF-α (pg.ml)**

**Statistics**

| **Variable** | **Group** | **Mean** | **SE Mean** |
| --- | --- | --- | --- |
| TGF-β (pg.ml) | MXF 15mg/kg | 403.1 | 30.6 |
|  | MXF 3.75mg/kg | 232.0 | 20.8 |
|  | MXF 7.5mg/kg | 358.1 | 28.0 |
|  | Negative Control | 253.3 | 14.2 |
|  | Positive Control | 383.5 | 36.9 |
|  |  |  |  |
| IL-10 (pg.ml) | MXF 15mg/kg | 17.570 | 0.000000 |
|  | MXF 3.75mg/kg | 43.02 | 3.32 |
|  | MXF 7.5mg/kg | 30.78 | 7.46 |
|  | Negative Control | 20.345 | 0.790 |
|  | Positive Control | 48.205 | 0.958 |
|  |  |  |  |
| IL-6 (pg.ml) | MXF 15mg/kg | 8.5700 | 0.000000 |
|  | MXF 3.75mg/kg | 13.783 | 0.391 |
|  | MXF 7.5mg/kg | 11.21 | 1.33 |
|  | Negative Control | 15.628 | 0.882 |
|  | Positive Control | 13.572 | 0.265 |
|  |  |  |  |
| TNF-α (pg.ml) | MXF 15mg/kg | 20.990 | 0.000000 |
|  | MXF 3.75mg/kg | 35.49 | 1.09 |
|  | MXF 7.5mg/kg | 33.84 | 1.70 |
|  | Negative Control | 40.31 | 2.50 |
|  | Positive Control | 36.990 | 0.689 |
