# Supplementary material for: Understanding the impact of moxifloxacin on immune function: Findings from cytokine analyses and immunological assays in mice
Source: PLoS One. 2025 May 6;20(5):e0321961. doi: 10.1371/journal.pone.0321961 (PMC12054891; doi:10.1371/journal.pone.0321961)
Supplement: Attachment — Submitted filename: Responses to reviewers v2.docx [file pone.0321961.s005.docx]

Dear Editor,

We appreciate the constructive feedback provided by you and the reviewers. We have carefully addressed all the minor revisions as suggested. Below is our point-by-point response to each comment:

**Comment 1:** Review the reference list to ensure it is complete and correct. Remove retracted papers or provide a rationale for their inclusion. If cited, mark retracted references appropriately.

**Response:** We have thoroughly reviewed our reference list. No retracted references were included in our manuscript.

**Comment 2:** Add the date of approval of the Animal Ethics Committee.

**Response:** The date of approval from the Animal Ethics Committee has been added in the manuscript.

**Comment 3:** Proofread the manuscript for minor language corrections.

**Response:** We have carefully proofread the manuscript and corrected minor language issues.

**Comment 4:** Avoid repeated use of "Moxifloxacin," as the abbreviation "MXF" is sufficient. Use the full form only once in the abstract and body, then "MXF" throughout. Avoid starting sentences with abbreviations and capitalize words appropriately.

**Response:** We have revised the manuscript to ensure that "Moxifloxacin" appears in full form only the first time in the abstract and body followed by "MXF" throughout.

Sentences have been rewritten to avoid starting with abbreviations and capitalization inconsistencies have been corrected.

**Comment 5:** In line 322 of the last paragraph of the discussion, add a space after a period.

**Response:** The formatting issue has been corrected by adding the necessary space.

**Comment 6:** Ensure consistency in figures, including uniform fonts and labels. In Figure 1, standardize the use of “Control” vs. “control.”

**Response:** The figures have been revised. In Figure 1, "Control" has been used consistently.

**Comment 7:** Re-write the figure legends for grammatical correctness.

**Response:** The figure legends have been revised for grammatical accuracy and clarity.

We are thankful for the opportunity to improve our manuscript and thank you for your time and consideration.
